# Paget’s Disease of Bone and Normocalcemic Variant of Primary Hyperparathyroidism in an Osteoporotic Male: Exceptional Coexistence

**DOI:** 10.3390/reports8030180

**Published:** 2025-09-17

**Authors:** Ana-Maria Gheorghe, Oana Petronela Ionescu, Mihai Costachescu, Oana-Claudia Sima, Mara Carsote

**Affiliations:** 1 PhD Doctoral School of Carol Davila University of Medicine and Pharmacy, 010825 Bucharest, Romania; ana-maria.gheorghe@drd.umfcd.ro (A.-M.G.); oana-petronela.dirtu@drd.umfcd.ro (O.P.I.); carsote_m@hotmail.com (M.C.); 2 Department of Clinical Endocrinology V, “C.I. Parhon” National Institute of Endocrinology, 011863 Bucharest, Romania; 3 Department of Internal Medicine and Rheumatology, “Dr. Carol Davila” Central Military University Emergency Hospital, 010825 Bucharest, Romania; 4 Department of Radiology and Medical Imaging, “Dr. Carol Davila” Central Military University Emergency Hospital, 010825 Bucharest, Romania

**Keywords:** paget’s disease of bone, parathormone, calcium, osteoporosis, bone turnover marker, scintigraphy, tumor, case report

## Abstract

**Background and clinical significance**: Paget’s disease of bone involves anomalies of the bone metabolism; however, the presence of tumor-derivate abnormal parathyroid hormone (PTH) levels does not represent one of these disturbances. To our best knowledge, the association with normocalcemic variant of primary hyperparathyroidism has been limitedly reported, and here we introduce such an unusual overlap in a male suffering from osteoporosis. **Case presentation**: A 71-year-old, non-smoker man was hospitalized for mild, nonspecific dysphagia, asthenia, decreased appetite, and mild weight loss during the latest 2 months. His medical history included cardiovascular conditions and an abnormal PTH level with normal serum calcium under daily cholecalciferol supplements (tested twice during latest 12 months). The lab findings pointed out a normocalcemic primary hyperparathyroidism (PTH of maximum 163 pg/mL, and total calcium of 9.3 mg/dL) caused by a right parathyroid tumor of 1.2 cm, as confirmed by computed tomography (CT). Additionally, CT showed a left humerus lesion suggestive of Paget’s disease of bone, a confirmation that also came from the whole-body bone scintigraphy. The subject presented increased P1NP and osteocalcin, CrossLaps as bone formation, and resorption markers, with normal total alkaline phosphatase. CT scan also detected multiple vertebral fractures and small kidney stones. Zoledronate i.v. (3 mg, adjusted for creatinine clearance) was administered, taking into consideration all three bone ailments (Paget’s disease, high PTH/calcium, and osteoporosis) with further follow-up. **Conclusions**: This case highlights the following technical notes based on a real-life setting: 1. Despite the mentioned bone diseases, no bone pain was present. Loss of appetite, dysphagia, and asthenia may be a consequence of mineral metabolism disturbances. 2. The panel of blood bone turnover markers levels might be related to both hyperparathyroidism and Paget’s disease; notably, rare cases of Paget’s disease with normal alkaline phosphatase were prior reported. 3. A meticulous differentiation between secondary and primary hyperparathyroidism is required. In this instance, lack of hypocalcaemia and vitamin D deficiency was suggestive of the diagnosis of a primary variant. 4. Kidney stones, osteoporosis, and osteoporotic fractures may be correlated with both conditions, as well, while a dual perspective of the therapy, since the patient was not a parathyroid surgery candidate, included a first dose of zoledronate with consecutive long-term follow-up. To our best knowledge, the co-presence of normocalcemic variant of primary hyperparathyroidism represents an exceptional finding in a patient synchronously diagnosed with Pagetic lesions and osteoporosis complicated with vertebral fractures.

## 1. Introduction and Clinical Significance

Paget’s disease of bone is a chronic metabolic bone disorder marked by increased and disorganized bone remodeling, leading to deformities and fractures. It can manifest in two forms, mono-ostotic (a single bone is affected), or poly-ostotic (multiple bones are involved). The axial skeleton, particularly the pelvis, spine, skull, and proximal long bones are the most frequently affected sites [1,2]. Recent studies suggested a decline in the incidence of Paget’s disease of bone. Current prevalence is around 0.4% among adults. The prevalence increases with age, with estimations of 0.68% and 1.55% in individuals aged 55 years and older, being higher in men versus females [3,4].

The exact pathogeny is complex. Various potential mechanisms have been described, including genetic and environmental factors. Pathogenic variants in the *SQSTM1* gene, which encodes the p62 protein involved in autophagy, are frequently found in both familial and sporadic cases. Other genes such as *WNT16*, *RYR3*, *RYR1*, *NUP205*, *CAPN2*, and *NUP214* have also been found in association with Paget’s disease of bone. Environmental factors may include paramyxovirus, smoking, or lead exposure [5,6,7]. Patients are often asymptomatic and may be diagnosed incidentally through radiographic findings or elevated serum alkaline phosphatase levels. Symptomatic cases may experience bone pain, skeletal deformities, fractures, or complications such as secondary osteoarthritis and neurological compression syndromes. In rare cases, Paget’s disease of bone may undergo malignant transformation to osteosarcoma, chondrosarcoma, or fibrosarcoma [8,9,10].

The normocalcemic variant of primary hyperparathyroidism is a more recently defined form of primary hyperparathyroidism. It may be identified during extensive evaluation of patients with osteoporosis or kidney stones [11]. It is characterized by elevated parathyroid hormone (PTH) levels with normal albumin-corrected calcium, in the absence of secondary causes of hyperparathyroidism, such as vitamin D deficiency, medications influencing calcium metabolism, and chronic kidney disease. Moreover, these findings need to be consistent at two separate evaluations over 3 to 6 months [12].

Even though normocalcemic primary hyperparathyroidism is biochemically milder, and can be found in adults without any complications, evidence showed higher rates of complications, including osteoporosis and fractures, as well as kidney stones and gastrointestinal issues in these patients [13,14,15].

The indication of parathyroidectomy is a tailored decision based on individual complications and surgical risks, considering that guidelines neither indicate nor prohibit surgery in patients with normocalcemic primary hyperparathyroidism, but suggest that the criteria used for hypercalcemic patients could also be used in normocalcemic variant [16,17].

The aim of this report is to present an exceptional overlap between Paget’s disease of bone and normocalcemic primary hyperparathyroidism, two diseases which may impair bone metabolism in a male confirmed with osteoporosis.

## 2. Case Presentation

A 71-year-old male patient who does not smoke presented with nonspecific symptoms such as weight loss, dysphagia, asthenia, and loss of appetite over the past 2 months. His medical history included cardiovascular conditions (atrial fibrillation under anticoagulation therapy, congestive heart failure grade II NYHA, severe mitral regurgitation, and grade 2 aortic regurgitation). Also, he was recently detected with an abnormal parathyroid hormone (PTH) level. The patient presented normal serum calcium under daily cholecalciferol supplements that was repeatedly tested (as outpatient) during the latest 12 months. (Table 1) The family medical history was irrelevant.

On admission, the physical examination revealed a blood pressure of 110/66 mmHg and pulse of 61 bpm, a body mass index of 24.11 kg/m^2^, and mild kyphosis. The lab panel biologically confirmed a normocalcaemic variant of primary hyperparathyroidism, with normal vitamin D status and a creatinine clearance of 63 mL/min (Cockcroft–Gault Equation) and an estimated glomerular filtration rate (eGFR) of 78 mL/min/1.73 m^2^ [18], confirming a small degree of renal dysfunction (Table 1).

**Figure 1:**
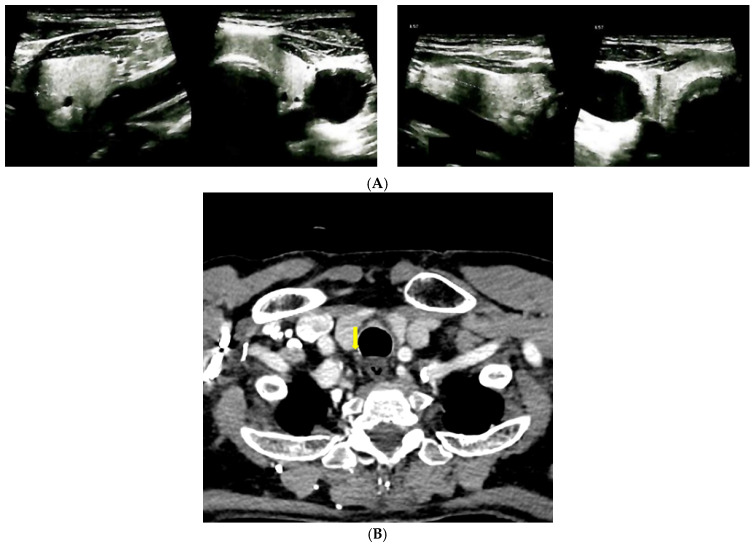

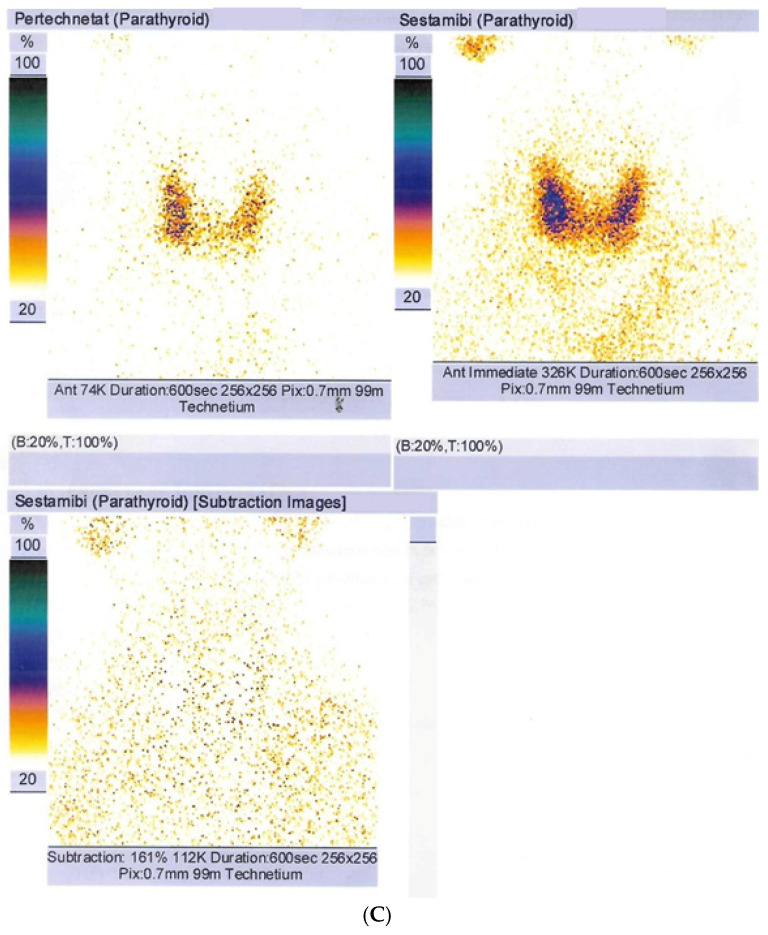
Imaging evaluation in a patient with Paget’s disease of bone and normocalcemic primary hyperparathyroidism. (**A**). Anterior neck ultrasound: left thyroid lobe of 1.01 × 2.2 × 3.32 cm, right thyroid lobe of 1.6 × 2.23 × 3.17 cm, homogeneous pattern and normal echogenicity in both thyroid lobes. (**B**). Contrast CT scan of the cervical area: right parathyroid tumor (yellow arrow) of 1.04 × 1.21 × 0.81 cm. (**C**). Planar images of Tc99m pertechnetate and Tc99m sestamibi subtraction scintigraphy: no tracer uptake.

The patient had increased bone turnover markers, both of formation (osteocalcin and P1NP) as well as serum CrossLaps as marker of resorption. Normal thyroid evaluation (Figure 1A) and ear, nose, and throat control was found.

Further investigations included i.v. contrast computed tomography (CT) which revealed a right parathyroid nodule suggestive for a parathyroid adenoma of 1.04 × 1.21 × 0.81 cm (para-tracheal, inferior to the right thyroid lobe). (Figure 1B).

A 99m Tecnetium (Tc) uptake was found negative at 99m Tc-pertechnetat (185 MBq)/99m Tc-sestamibi (740 MBq) parathyroid scintigraphy (effective estimated dose of 9.06 mSv) of the cervical and mediastinal areas (Figure 1C).

**Figure 2:**
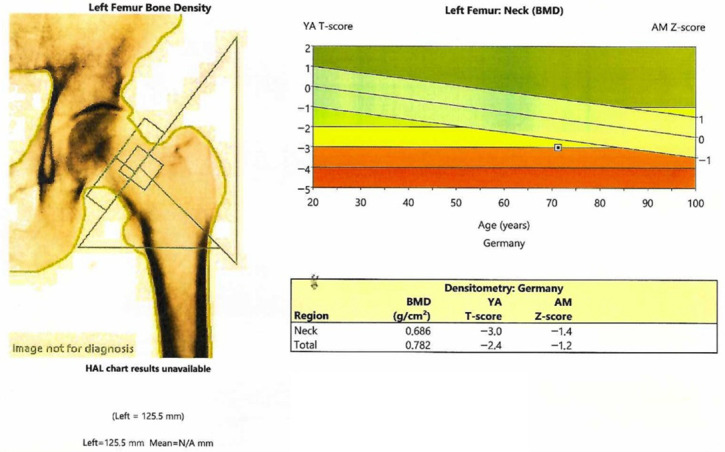
Left hip scan at central DXA.

The patient was confirmed with osteoporosis at central DXA (Dual-Energy X-Ray Absorptiometry; GE Lunar Prodigy device) associated with a partially degraded bone microarchitecture as reflected by the value of lumbar DXA-derivate trabecular bone score (TBS) of 1.240 (Table 2, Figure 2).

**Figure 3:**
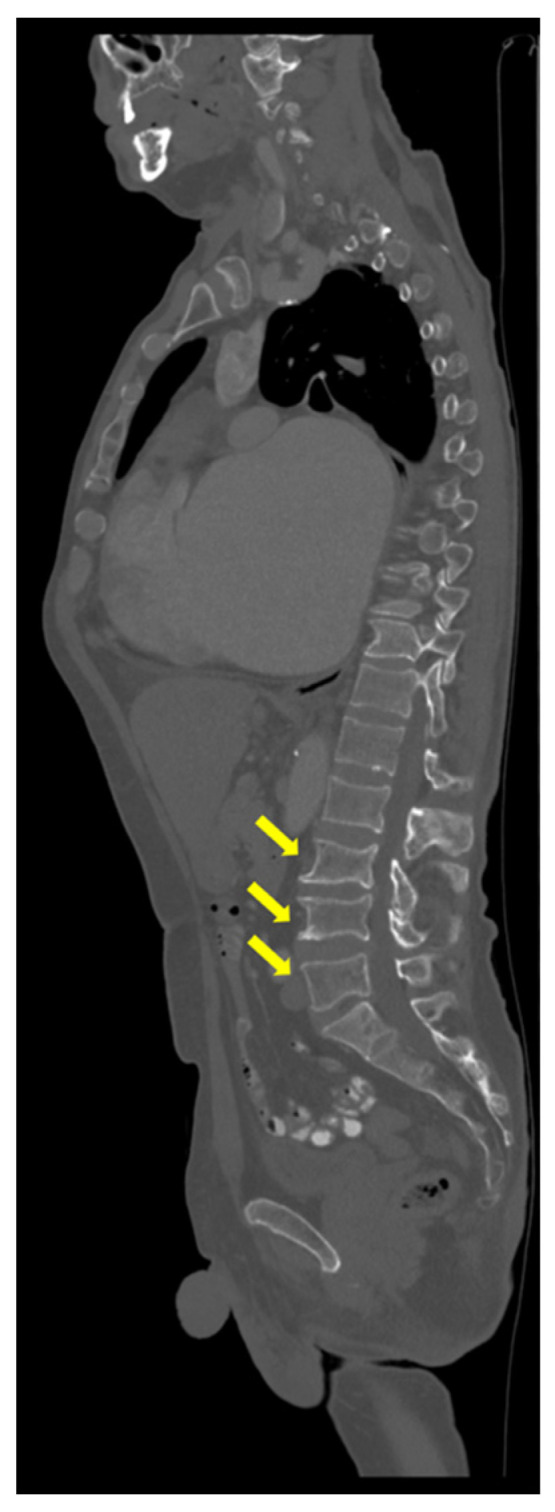
Native CT showing L3, L4, and L5 vertebral fractures (yellow arrow).

**Figure 4:**
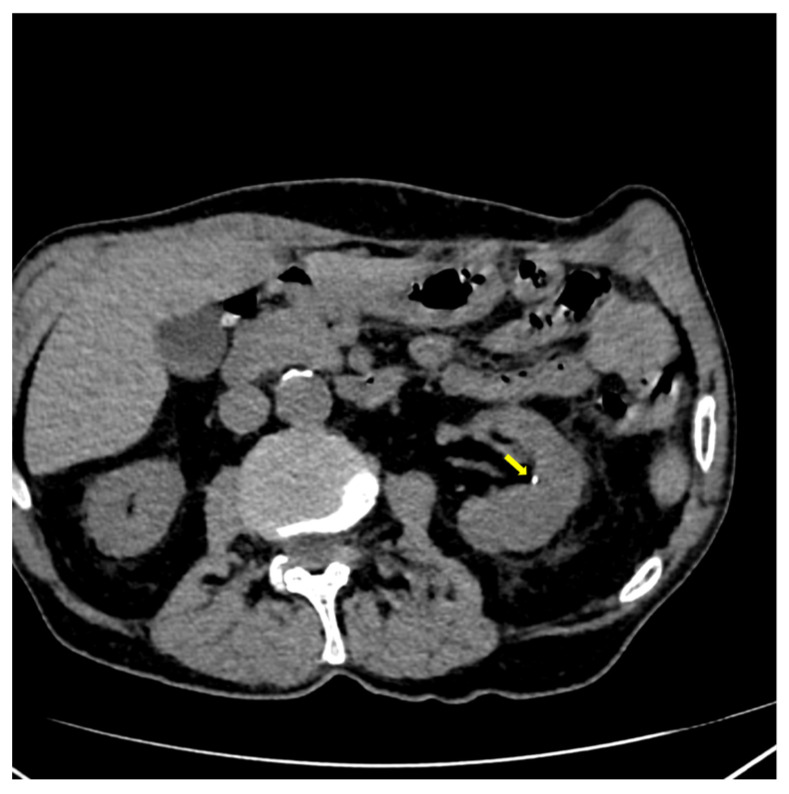
CT showing micro-calculi (yellow arrow) in the middle calyx of the left kidney.

Also, CT scan detected multiple mild vertebral fractures (yellow arrow) at L3, L4, and L5 lumbar vertebras (Figure 3), and bilateral micro-calculi in the upper calyx of the right kidney and the middle calyx of the left kidney (Figure 4).

**Figure 5:**
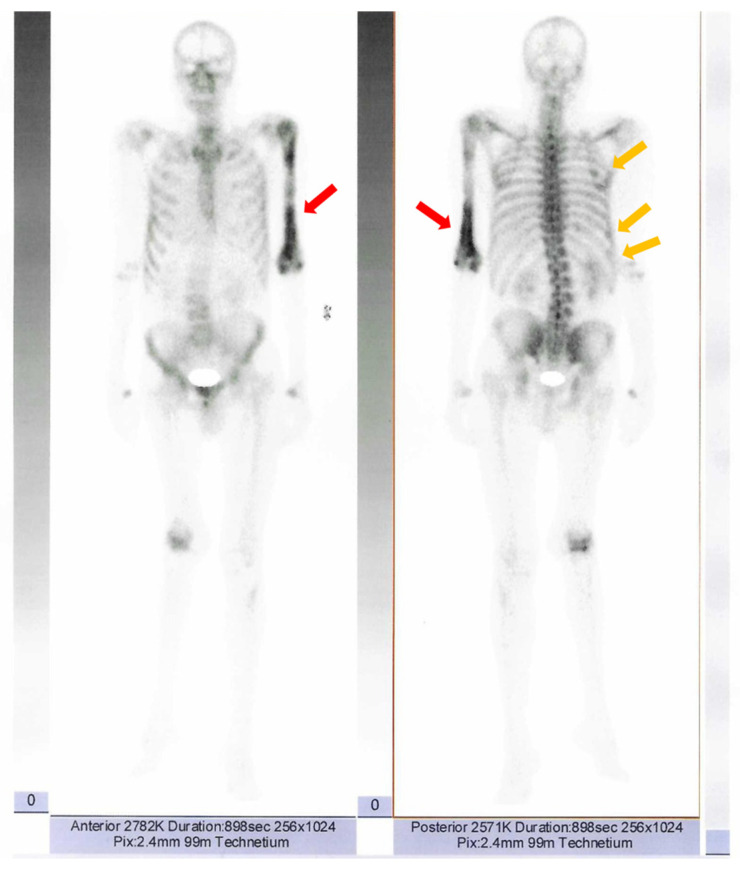
Planar images of Tc99m whole-body scintigraphy showing increased and heterogeneous uptake of the radiotracer at the left humerus (red arrow), more intense in the distal half, predominantly in the cortical bone and moderately active foci projected at the level of the right posterior costal arches of third, seventh, and eighth ribs (orange arrows).

**Figure 6:**
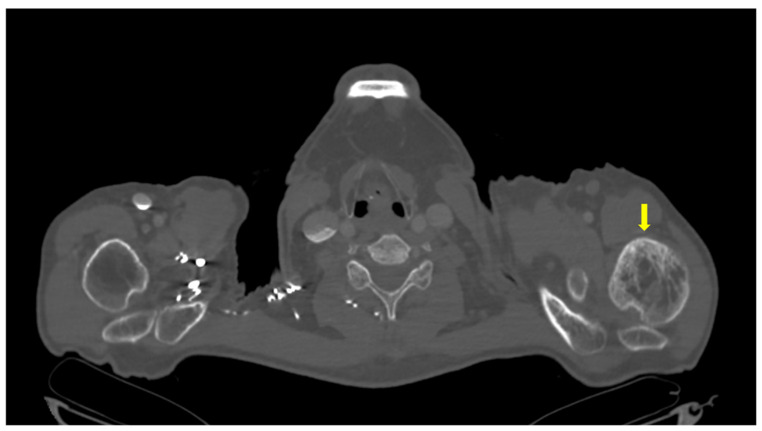
Contrast CT showing Paget’s disease of bone lesion at the left humerus (yellow arrow).

**Figure 7:**
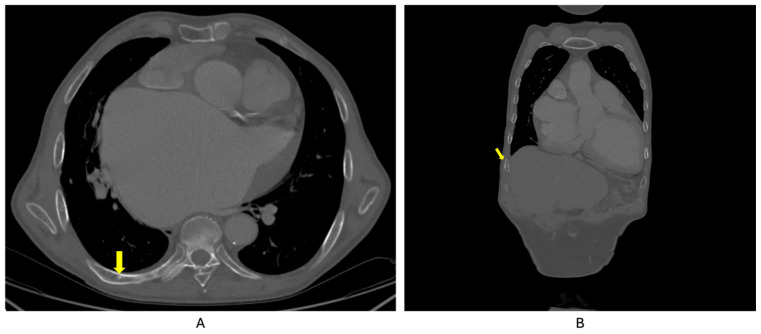
CT showing (**A**). Pagetic lesions (yellow arrows) of the 7th rib in the axial plane; (**B**). Pagetic lesion of the 7th rib in the coronal plane.

Whole-body 99m Tc bone scintigraphy revealed increased and heterogeneous uptake of the radiotracer at the left humerus, predominantly in the cortical bone, suggestive for Paget’s disease of the bone (Figure 5). The lesion was also confirmed at CT as well (Figure 6). In addition, moderately active foci projected at the level of the right posterior costal arches of third, seventh, and eighth ribs were identified (Figure 7).

The patient was diagnosed with normocalcemic primary hyperparathyroidism and Paget’s disease of the bone. Additionally, due to gastrointestinal complaints, the patient also underwent an upper gastrointestinal endoscopy, which revealed Los Angeles grade A esophagitis and atrophic antral gastritis.

Considering the high cardiac risk, parathyroid surgery was postponed. The patient continued cholecalciferol supplementation (2000 UI/day) and received intravenous zoledronate 3 mg. The re-administration at 6 to 12 months will depend on the outcome, particularly the bone turnover markers profile, since further follow-up is mandatory, including deciding the long-term regime.

## 3. Discussion

This case highlights the following technical notes based on a real-life setting.

### 3.1. Clinical Manifestations in Ailments of the Bone Metabolism

The patient was initially admitted for loss of appetite, asthenia, without bone/muscle complains. Primary hyperparathyroidism may present with muscle weakness even in normocalcemic variants [19,20]. Other frequent complaints are fatigue, reported in 48% of patients [21], and anorexia [22]. Dysphagia has also been reported in patients with primary hyperparathyroidism, either linked to hypercalcemia [23], or to non-functioning parathyroid tumors that suffered hemorrhage/necrosis [24,25,26]. On the other hand, patients with Paget’s disease of bone frequently experience localized bone pain due to deformities, micro-fractures, and osteoarthritis [6]. Bone pain is a common finding in primary hyperparathyroidism as well [27]. In spite of the frequency of this clinical manifestation in both conditions, the patient did not complain of bone pain. Another complication occurring both in Paget’s disease of bone and primary hyperparathyroidism is nephrolithiasis, and the subject was confirmed at CT scan with this complication [28]. Of note, a component of secondary hyperparathyroidsim cannot be entirely ruled out (and it might be, in fact, overlapped), but there was only a small degree of renal dysfunction and the complete correction of vitamin D deficiency did not explain the persistent high PTH values.

### 3.2. Bone Turnover Markers

In this case, the blood bone turnover markers profiles showed an increased level of bone formation markers [osteocalcin and procollagen type 1 amino-terminal peptide (P1NP)] and bone resorption marker (CrossLaps) with normal total alkaline phosphatase (ALP). The hallmark of Paget’s disease of bone is increased and disorganized bone remodeling. This is biochemically reflected in the elevated levels of bone turnover markers. High bone formation is captured by the increased serum total ALP, bone-specific ALP (of note, this was not available in this instance), P1NP, and osteocalcin, while resorption is typically showed by high urinary N-telopeptide (uNTx) and type I collagen C-telopeptide (CTX) [29,30].

Guidelines recommend the use of serum total ALP, together with liver function tests, as biochemical screening. The assessment of bone-specific ALP, P1NP, and uNTx levels could identify disease activity in cases of high clinical suspicion with normal serum total ALP [31]. Even though serum total ALP is a sensitive test, patients with monostotic disease may have normal ALP levels. In such case, P1NP and bone-specific ALP are more sensitive and specific markers [32]. More than being a diagnostic tool, bone turnover markers, especially P1NP, are useful in monitoring the disease activity and treatment response, as shown by a meta-analysis which found a correlation between P1NP and scintigraphy-based activity following therapy (r = 0.674, *p* = 0.000) [33].

In primary hyperparathyroidism, both resorption and formation occur on a higher bone surface generating increased release of degradation products and newly formed proteins, resulting in elevated levels of both resorption and formation markers [34]. While hypercalcaemic variant is associated with high bone turnover markers levels, studies regarding normocalcemic type showed rather inconclusive results. For instance, a prospective study on 104 patients with primary hyperparathyroidism revealed higher P1NP (73.5 versus 49.2 ng/mL, *p* = 0.005), osteocalcin (37.4 versus 23.5 ng/mL, *p* = 0.02), and CTX levels (0.68 versus 0.38 ng/mL, *p* = 0.001) in hypercalcaemic versus normocalcemic variant [35]. On the other hand, another study on 316 individuals confirmed with primary hyperparathyroidism reported lower ALP levels in normocalcemic patients (86.9 versus 94.1, *p* = 0.016) but found no statistically significant difference in terms of CTX (*p* = 0.483) [15].

### 3.3. Additional Pitfalls: Secondary Hyperparathyroidism in Paget’s Disease of Bone

The interplay between calcium levels, vitamin D status, and PTH in Paget’s disease of bone is complex and derives from the accelerated bone turnover and the erratic bone formation caused by the abnormal activity of osteoclasts and the induction of bone formation, as well as a malfunctioned interaction between osteoclasts and osteoblasts [36,37]. The increased calcium demands during the formation phase may deplete calcium levels, stimulating the parathyroid hormone secretion, leading to secondary hyperparathyroidism [38], further accentuated by vitamin D deficiency caused by excessive bone utilization [39]. In this instance, the patient had no vitamin D deficiency and did not experience hypocalcemia, and this was useful for the biological confirmation of primary hyperparathyroidism in accordance with the identification of a parathyroid tumor at CT scan. Of note, for small adenomas, 99m Tc scintigraphy might not confirm the tracer uptake.

### 3.4. Complications of Normocalcemic Primary Hyperparathyroidism

In this case, vertebral fractures represented complications of osteoporosis, while low bone mineral density is mostly attributed to the parathyroid disease. Normocalcemic primary hyperparathyroidism associates a less affected biochemical profile compared to hypercalcaemic type. However, complications such as osteoporosis and kidney stones are frequent in both types [16,40]. Moreover, in surgery candidates, normocalcemic subjects may have even a higher rate of kidney stones compared to hypercalcaemic patients (53.3% versus 30.4%, *p* < 0.001) [41]. While in patients without any bone or kidney complications, normocalcemic variant was reported with a small prevalence of 0.74% [14], a selection bias should be taken into consideration, as individuals with normocalcemic primary hyperparathyroidism are often identified during extensive investigations for osteoporosis, fragility fractures, or kidney stones [13].

### 3.5. A Dual Perspective of the Case Management

In this instance, the presence of a high cardiovascular risk excluded the patient as a surgery candidate. Generally, the curative treatment for primary hyperparathyroidism is parathyroidectomy. According to guidelines, classical complications such as osteoporosis, fragility fractures, and kidney stones represent surgery indications. However, data regarding non-classical complications such as the co-presence of metabolic and cardiovascular disease are insufficient to recommend a parathyroid surgery. While there are no distinct recommendations for parathyroid surgery in normocalcemic variant, the current guidelines apply similar indications as for hypercalcaemic patients [12,17,42,43]. Preoperative localization is essential for an adequate surgical management. However, in normocalcemic type, the tumor localization was reported to be more difficult than in hypercalcemia variant, with more frequent discordances between different imaging techniques (e.g., CT, 99m Tc scintigraphy, ultrasound, etc.) [44,45,46]. In addition, a small adenoma size may not be localized, which represents a bias of detection as well [12,17]. Surgical decision is therefore tailored for each patient. Taking into consideration the presence of osteoporosis and kidney stones, the patient met the surgical criteria. However, given the parathyroid tumor localization, as well as the high cardiovascular risk, the patient was managed conservatively. Long-term follow-up of calcium levels and complications (e.g., osteoporosis, cardiovascular status) is mandatory. Generally, male osteoporosis, even rarer than that found in women, may be related to secondary causes such as glucocorticoid excess, alcohol consumption, systemic diseases, malignancies, as well as endocrine disorders, including hypogonadism and hyperparathyroidism, both variants, with hypercalcaemic and normocalcemic being reported to involve a low bone mineral density and a higher osteoporotic fracture risk than detected in age-matched general population [47,48,49].

Current guidelines recommend the use of bisphosphonates, particularly zoledronate, to reduce the metabolic activity of Paget’s disease of bone and to control the symptoms [50]. Another treatment option, although off-label, is denosumab [51] in patients with contraindications [52,53,54] or adverse reactions to bisphosphonates [55], and with giant cell tumor of bone [56,57,58]. In this case, zoledronate was chosen to improve the bone status from a dual perspective of both conditions. Further decision to prolong the interval until the following administration for 6 up to 12 months or more is based on the clinical evolution and lab biomarkers, particularly, bone turnover profile, as well as bone imaging for Pagetic lesions.

### 3.6. Similar Cases in the Literature

Paget’s disease of bone and (hypercalcemic) primary hyperparathyroidism is a very rare association, and a limited number of studies have investigated this association. For instance, according to one cross-sectional multicentric study, patients with Paget’s disease of bone have higher rates of primary hyperparathyroidism compared to controls (*p* < 0.01) [28]. On the other hand, another study reported a prevalence of primary hyperparathyroidism of 4.4% in subjects with Paget’s disease of bone [59]. In addition, there are a few case reports of patients suffering from both diseases [60,61,62,63,64,65,66,67], but to the best of our knowledge, we identified only a similar report with coexisting normocalcemic primary hyperparathyroidism and Paget’s disease of bone. This case, published by Stathopoulos et al. [68], involved a 74-year-old male who initially presented for hip pain. He was diagnosed with Paget’s disease of bone based on the imaging findings. The patient had elevated PTH levels despite normal serum calcium, and no secondary causes of hyperparathyroidism were identified. Ultrasonography identified a parathyroid tumor and he underwent a selective parathyroidectomy [68].

## 4. Conclusions

This case in point shows an uncommon overlapping circumstance of Paget’s disease of bone and normocalcemic primary hyperparathyroidism; both ailments being contributors to a low bone mineral density and kidney stones, in addition to a higher level of bone turnover markers. Normocalcemic primary hyperparathyroidism is increasingly recognized due to more frequent screening of serum calcium assays and bone turnover markers. The clinical significance of this coexistence also includes the fact that Paget’s disease of bone might aggravate a secondary component of hyperparathyroidism or mask a hypercalcemia caused by a parathyroid tumor; an increased fracture risk might be related to both conditions, while the impairment of the renal function may restrict the use of bisphosphonates across lifespan. In this particular instance, zoledronate was considered helpful for both conditions, while further decision of re-administration requires a meticulous analysis of bone turnover and imaging lesions.

## Figures and Tables

**Table 1:** Lab panel in a 71-year-old male adult admitted for dysphagia, loss of appetite, and asthenia. Bold means abnormal values.

Parameter (Unit)	Pre-Admission (Within 12 Months)	On Current Admission	Normal Range
**Mineral Metabolism**			
Serum total calcium (mg/dL)	9.36, repeated 9.79	9.3, repeated 8.9	8.4–10.2
Serum ionized calcium (mg/dL) ***		4.11, repeated 3.98	3.9–4.9
Phosphorus (mg/dL)		3.2, repeated 3.69	2.3–4.7
Total proteins (g/dL) ***		7, repeated 6.4	6.4–8.6
PTH (pg/mL)	**135.8**, repeated **256.6** *	**163.7**, repeated **109.4**	17.3–74.1
25-hydroxyvitamin D (ng/mL)	36.61, repeated 48 **	36.6 **	20–100
24 h urinary calcium (g/24 h)		0.07	0.07–0.3
Alkaline phosphatase (U/L)		84	40–150
Osteocalcin (ng/mL)		**66.93**	14–46
CrossLaps (ng/mL)		**1.72**	0.118–0.776
P1NP (ng/mL)		**132.5**	20.25–76.31
**Non-Mineral Metabolism**			
Creatinine (mg/dL)		1.03	0.5–1.2
Urea (mg/dL)		61	18–55
eGFR (mL/min/1.73 m^2^)		78	Normal (Stage 1): >90
			Stage 2: 60–89
			Stage 3a: 45–59
			Stage 3b: 30–44
			Stage 4: 15–29
			Stage 5: <15
Fasting glycaemia (mg/dL)		99.8	74–106
ALT (U/L)		23	0–55
AST (U/L)		23	5–34
TSH (µIU/mL)		4.09	0.35–4.94
Free T4 (pmol/L)		14.89	9–19

Abbreviations: ALT = aspartate aminotransferase; AST = alanine transferase; eGFR = estimated glomerular filtration rate; Free T4 = free thyroxine; P1NP = procollagen type 1 N-terminal propeptide; PTH = parathyroid hormone; TSH = thyroid stimulating hormone; * PTH normal ranges: 12–88 pg/mL; ** under daily cholecalciferol 2000 UI; *** ionized calcium formula of calculation = (6 × serum total calcium (mg/dL)—total proteins (g/dL)/3)/(total proteins (g/dL) + 6); detection methods for 25-hydroxyvitamin D, PTH, and bone turnover markers—electrochemiluminescence), for total calcium—spectrophotometry.

**Table 2:** DXA showing osteoporosis (GE Lunar Prodigy).

	BMD (g/cm^2^)	T-Score	Z-Score
L1-L4 Lumbar	0.882	−2.9	−1.8
Femoral neck	0.686	−3.0	−1.4
Total hip	0.782	−2.4	−1.2
Third distal radius	0.811	−1.8	−1.0

Abbreviations: BMD = bone mineral density.

## Data Availability

The original data presented in the study are included in the article, further inquiries can be directed to the corresponding authors.
